# A validated model for predicting live birth after embryo transfer

**DOI:** 10.1038/s41598-021-90254-y

**Published:** 2021-05-24

**Authors:** Michael S. Awadalla, Kristin A. Bendikson, Jacqueline R. Ho, Lynda K. McGinnis, Ali Ahmady

**Affiliations:** Division of Reproductive Endocrinology and Infertility, Department of Obstetrics and Gynecology, Keck School of Medicine, University of Southern California, LAC+USC Medical Center, 2020 Zonal Avenue, IRD Room 533, Los Angeles, CA 90033 USA

**Keywords:** Computational biology and bioinformatics, Developmental biology, Embryology

## Abstract

Accurately predicting the probability of live birth and multiple gestations is important for determining a safe number of embryos to transfer after in vitro fertilization. We developed a model that can be fit to individual clinic data for predicting singleton, twin, and total live birth rates after human embryo transfer. The predicted and observed rates of singleton and twin deliveries were compared in a tenfold cross-validation study using data from a single clinic. The model presented accounts for patient age, embryo stage (cleavage or blastocyst), type of transfer cycle (fresh or frozen) and uterine/universal factors. The standardized errors for rates of singleton and twin deliveries were normally distributed and the mean errors were not significantly different from zero (all *p* > 0.05). The live birth rates per embryo varied from as high as 43% for fresh blastocysts in the 35-year-old age group to as low as 1% for frozen cleavage stage embryos in the 43-year-old age group. This quantitative model or a simplified version can be used for clinics to generate and analyze their own data to guide the number of embryos to transfer to limit the risk of multiple gestations.

## Introduction

Although the goal of in vitro fertilization (IVF) and other fertility treatments is a single healthy intrauterine pregnancy and delivery, twins and higher order multiples are a frequent occurrence. According to the 2016 Center for Disease Control (CDC) Assisted Reproductive Technology (ART) National Summary Report, of the 19,137 live deliveries from ART cycles using fresh embryos with fresh nondonor eggs, 18.8% were twin deliveries and 0.6% were triplets or more^1^. Multiple gestations increase maternal and fetal morbidity and mortality not only through preterm labor and delivery but also preeclampsia, gestational diabetes, and other pregnancy complications^2^. A recent American Society for Reproductive Medicine (ASRM) practice committee document gives recommended limits to the number of embryos to transfer and encourages individual clinics to use their own data in order to minimize multiple gestations^3^.

Many previous models have been developed to guide the number of embryos to transfer^4–10^. Typical models build on the Speirs model which assumes that embryos implant independently when uterine factors are favorable^4^. Evidence supports the main assumption of the Speirs model that an individual embryo’s probability of implantation and live birth is not directly impacted by other embryos but there are universal factors that affect all embryos transferred concurrently^4,9,11,12^. These factors have been described in both general and specific terms such as “uterine receptivity”, “endometrial receptivity”, and “transfer efficiency”^4,5^. If universal factors are favorable all embryos are more likely to implant than if universal factors are not favorable. Taking universal factors into account results in higher predicted rates of multiples and lower predicted rates of singleton pregnancy than would be expected if embryos simply combined according to individual embryo implantation rates (as if each embryo is modeled as a coin flip). This concept of universal factors is supported by previous research that found higher rates of multiple pregnancy than predicted by a model which did not account for universal factors^9^.

Despite the availability of these models, routine use of prediction models to guide the number of embryos to transfer is not practiced and current guidelines are based on general rather than individual clinic data^3^. This begs the question: why have these models not been adopted for use in clinical practice? Several possible explanations include complexity that limits implementation, the information provided in some studies is not clinically useful, rapidly advancing IVF technology outdates models quickly, and reluctance to use a model that is not easy to understand. There is clearly a need for a simple, accurate, and quantitative model that can be updated with current data and easily applied to individual clinic data.

Single embryo transfer (SET) is a great option to limit the risk of multiple pregnancies but carries important limitations. Some patients may desire multiple embryo transfer for cost effectiveness, after failing one or more SETs, or for a cleavage stage embryo transfer. According to the CDC 2016 ART National Summary Report, the average number of embryos transferred for different age groups ranged from 1.4 to 1.6 embryos in autologous frozen embryo transfer (FET) cycles and from 1.5 to 2.4 embryos in autologous fresh cycles^1^. Preimplantation genetic testing for aneuploidy (PGT-A) has aided in selecting embryos for SET but has limitations including cost, embryologist expertise required to perform a trophectoderm biopsy, turnaround time which makes fresh embryo transfer logistically challenging, and possible embryo damage from the biopsy. Given the fact that it took many years to determine that cleavage stage biopsies decrease implantation potential^13^, it is possible that the same may be discovered for blastocyst biopsy. Although a recent non-selection study did not detect a decreased sustained implantation rate after blastocyst biopsy^14^, results from a recent multicenter randomized clinical trial suggest that this is likely not the case at all clinics^15^. The recent multicenter trial showed no difference in ongoing pregnancy rate after single frozen embryo transfer based on selection by PGT-A versus morphology of unbiopsied blastocysts (50% vs 46%). This seems to indicate that blastocyst biopsy either decreased the live birth rate of an embryo, that viable embryos were not classified as euploid, or a combination of both. Blastocysts with mosaic and aneuploid biopsy results have indeed been shown to occasionally result in healthy liveborn children^16–18^. Additionally, the long-term effects of embryo biopsy on human growth and development are unknown. Thus, there is still a role for transferring untested cleavage and blastocyst stage embryos. The ability to use one's own clinic data to guide the number of untested embryos to transfer is pertinent to modern practice.

The primary objective of this study is to develop and validate a practical model based on individual clinic data for predicting singleton, twin, and total live birth rates after IVF with transfer of one or more untested embryos. Additional objectives include demonstrating how a model can be fit to individual clinic data, reporting the results of fitting the model to our clinic data, providing a framework for future studies to include embryo morphology in a model, and to provide the necessary methods and computer code to fit the model to other datasets.

## Materials and methods

### Study population

**Figure 1:**
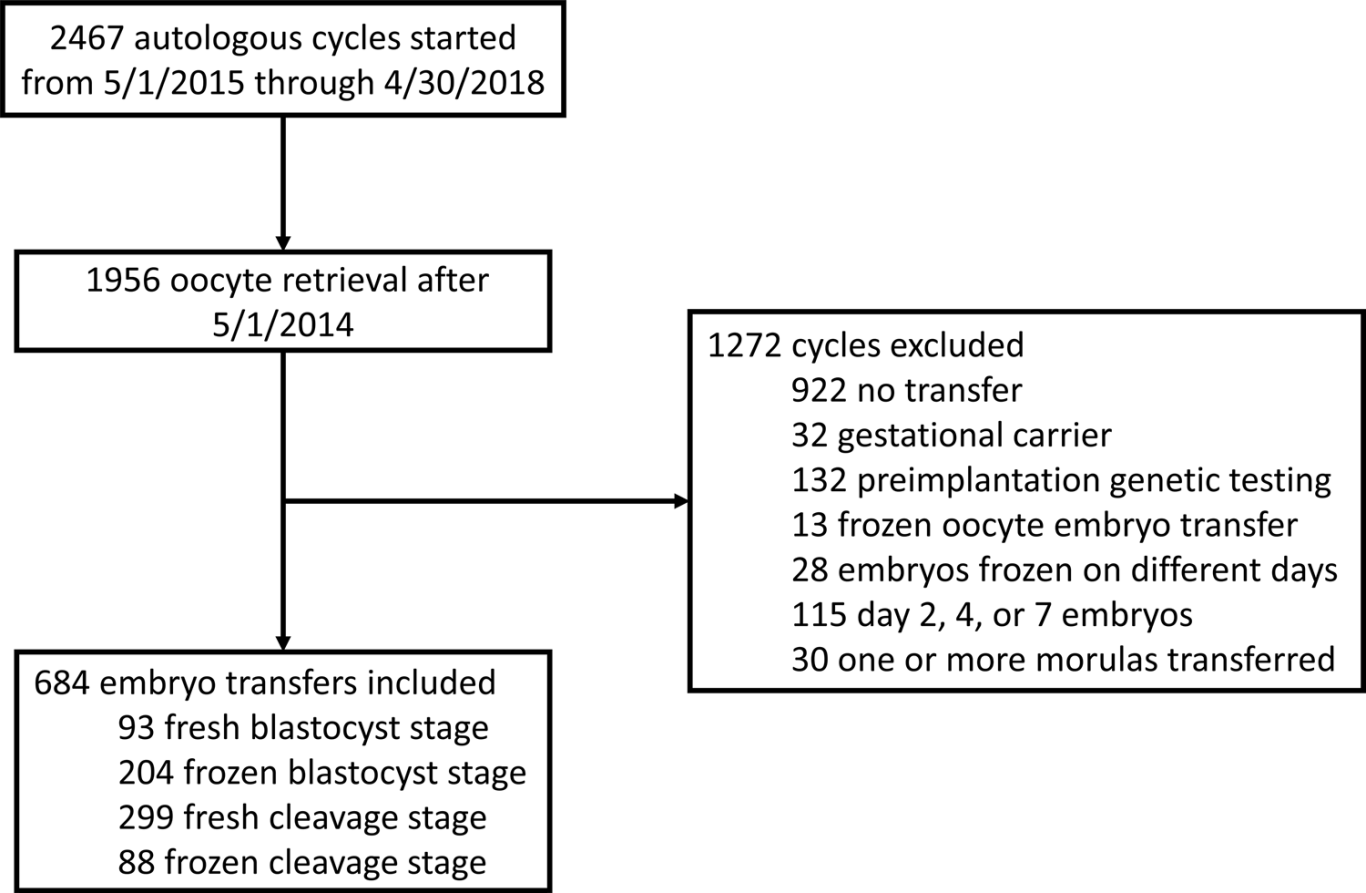
Embryo transfer cycles included and excluded.

Patients undergoing IVF at a single center from May 2015 through April 2018 were included in the study. Day 3 embryos were included in the cleavage stage group while day 5 and day 6 embryos were included in the blastocyst stage group. Exclusion criteria were no transfer, gestational carrier, preimplantation genetic testing, frozen oocyte embryo transfer, embryos frozen on different days, day 2/4/7 embryo transferred, and one or more morulas transferred (Fig. 1). Our analysis included 684 embryo transfers of a total of 1675 embryos for an average of 2.4 embryos per transfer. All of the data was collected prospectively in real time. Demographics and cycle characteristics are included in Supplemental Table S1.

### Embryo transfer model

**Figure 2:**
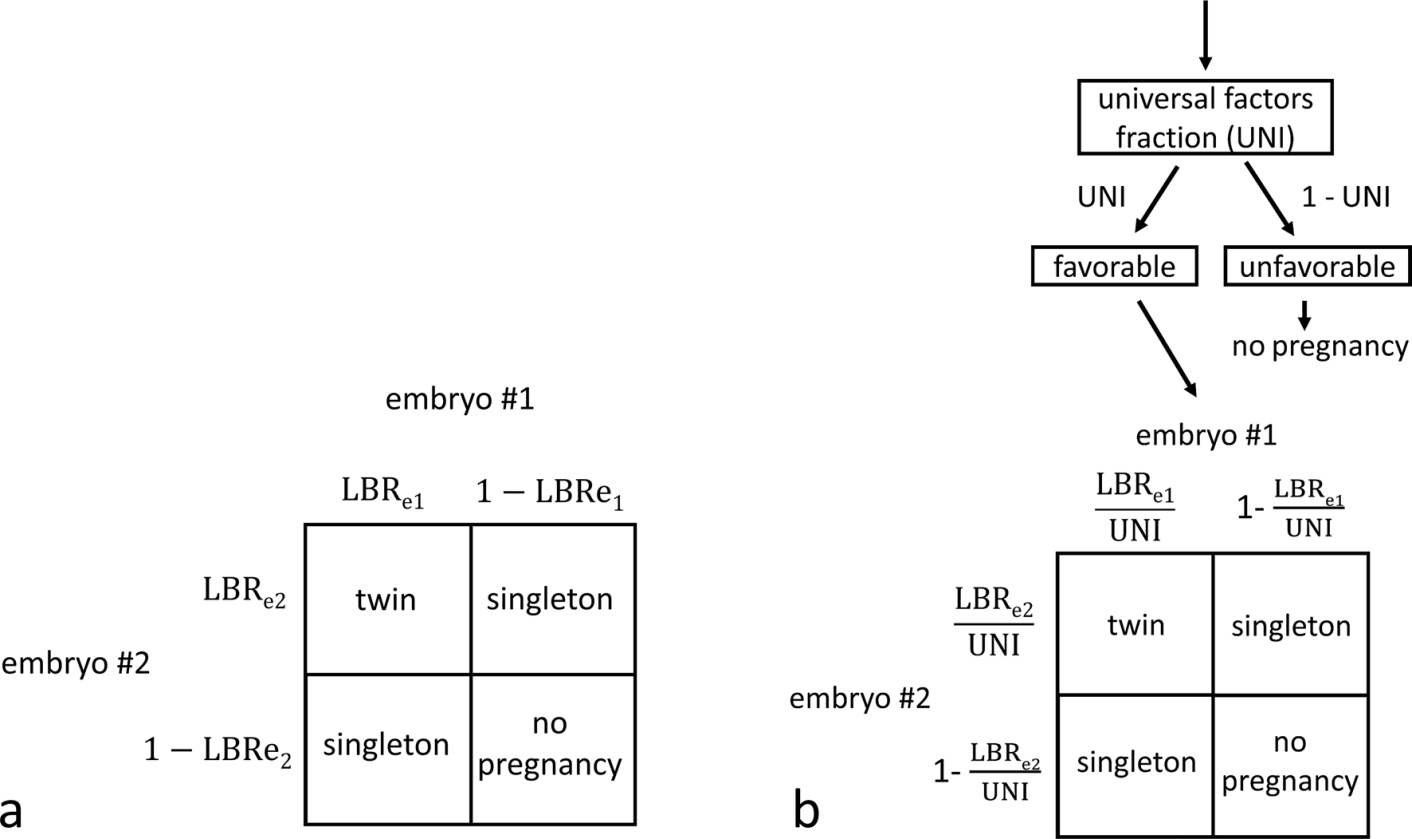
Embryo transfer models demonstrated for transfer of two embryos. (**a**) Demonstrates logic for independent embryo implantation and (**b**) demonstrates logic for independent embryo implantation only when universal factors are favorable. The universal factors fraction is a fraction from 0 (never favorable) to 1 (always favorable). The probability of each outcome is equal to the product of the terms next to the corresponding arrows above and the corresponding terms on the perimeter of the square. For example, the predicted probability of twins in panel b is UNI × $\frac{{{\text{LBRe}}1{ }}}{{{\text{UNI}}}}$ × $\frac{{{\text{LBRe}}2{ }}}{{{\text{UNI}}}}$. This same logic can be applied to transfer of more than two embryos. UNI, universal factors fraction; LBR_e1_, live birth rate for embryo 1; LBR_e2_, live birth rate for embryo 2. This figure is being reproduced from a previous publication^19^.

Our embryo transfer model is defined by the logic in Fig. 2 which is demonstrated for a transfer of two embryos. This logic can be easily extended to transfer of any number of embryos. Rather than each embryo implanting independently as shown in Fig. 2a, we assume that embryos implant independently only when universal factors are favorable as shown in Fig. 2b. To apply the embryo transfer model to predict outcomes, the live birth rate (LBR) for each embryo and universal factors fraction (UNI) for each transfer must be known. The LBRs are represented as fractions between 0 and 1. UNI is represented as a fraction between 0 (never favorable) and 1 (always favorable). By the logic in Fig. 2a the predicted rate of twins per transfer is LBR_e1_ × LBR_e2_ while the logic in Fig. 2b predicts a rate of UNI × $\frac{{{\text{LBRe}}1{ }}}{{{\text{UNI}}}}$ × $\frac{{{\text{LBRe}}2{ }}}{{{\text{UNI}}}}$ which simplifies to $\frac{{{\text{LBRe}}1{ } \times {\text{LBRe}}2{ }}}{{{\text{UNI}}}}$. The model shown in Fig. 2b simplifies to that of Fig. 2a in two scenarios: when only one embryo is transferred and when universal factors are always favorable (UNI = 1). The assumptions and logic in Fig. 2b are used in this analysis.

### Determination of live birth rates

Live birth rates (LBRs) per embryo were determined by age at oocyte retrieval and four categories of embryo transfer: fresh cleavage stage, frozen cleavage stage, fresh blastocyst stage, and frozen blastocyst stage. Patients aged 25–34 years were analyzed as a single group. For patients aged 35 years old and greater, live birth rates for an individual age were based off of a five-year group of patients centered on the age of interest. The analysis was performed this way to smooth out random variation in the data. For example, all embryo transfer data from patients aged 33–37 years was used to generate live birth rates for patients 35 years old. Each embryo transfer was modeled as an equation as follows: number of embryos transferred × LBR = number of live births that resulted. The least squares solution for LBR was determined using linear algebra for all the transfers in a given age group and transfer category combination with the use of MATLAB (version 9.5, MathWorks). This analysis was also performed using ongoing pregnancy (defined as detection of a fetal heartbeat at 6–8 weeks gestation) as the outcome.

### Calculation of universal factors fraction

A computer simulation was performed to determine the best fit universal factors fraction (UNI) based on LBRs per embryo and actual transfer live birth outcomes using the logic in Fig. 2b ^20^. This was done by assuming that if universal factors are not favorable then no live births occur. However, if universal factors are favorable then each embryo behaves independently of the others and results in a live birth at a rate of $\frac{{{\text{LBR}}}}{{{\text{UNI}}}}$. A best fit UNI was determined separately for fresh and frozen embryo transfers. The UNI was determined to best fit the number of observed singleton live births that resulted from multiple embryo transfers. The best fit solution was determined using a custom computer program in MATLAB which estimates the likelihood of outcomes of multiple embryo transfers for varying values of UNI ^20^. Values of UNI closer to 1 correspond to higher rates of singleton pregnancy and delivery. UNI values were also determined for transfer subgroups and using ongoing pregnancy as the outcome.

### Validation of the model and statistical analysis

To validate the model, a tenfold cross-validation study was performed separately for fresh and frozen embryo transfers using a custom computer program in MATLAB. Each embryo transfer was assigned to one of ten groups using a random number generator. To predict the outcomes of group 1, groups 2 through 10 were used as training data to determine best fit rates of live birth for each age and embryo transfer category combination. Then the best fit UNI values were determined. This analysis was repeated 10 times, each time excluding a different group. A computer simulation in MATLAB was used to perform bootstrapping based on the logic in Fig. 2b to determine predicted outcome probability distributions for rates of singleton, twin, and total live births per embryo transfer for the embryo transfers in the excluded group. Observed, predicted, and residual live birth rates were graphed for the best fit model as well as for a model that assumes universal factors are always favorable (UNI = 1, Fig. 2a) for comparison (Supplemental Fig. S1).

Univariate analysis was performed to evaluate the means and distribution of the standardized residual errors for the fresh and frozen transfer predicted singleton and twin delivery rates. Two-tailed Z-tests were used to evaluate the mean standardized errors and quantile–quantile plots were used to evaluate the error distributions. The mean standardized errors were not significantly different from zero for the fresh transfer predicted singleton delivery rate per transfer (*p* = 0.73) or twin delivery rate per transfer (*p* = 0.40). The mean standardized errors for frozen transfers were not different from zero for singleton delivery rate per transfer (*p* = 0.32) or twin delivery rate (*p* = 1.00). The observed standardized errors appeared to be approximately normally distributed on quantile–quantile plots (Supplemental Fig. S2). 95% confidence intervals (CIs) for the rates of live birth and ongoing pregnancy per embryo were determined using bootstrapping methods detailed in the supplemental methods section.

### Automation of data processing

We automate the process of data analysis so that data from an individual clinic (or multiple clinics) can be processed and live birth rates calculated quickly. Specifically, we use the “All Fields Export” feature in the clinic SART CORS database then extract pertinent cycles and cycle information with a SAS program (version 9.4, SAS Institute), and then perform the data analysis and validation studies through several custom MATLAB programs^20^. Since most of the analysis is automated, fitting a model to new data and validating the model takes only a few hours.

### Ethics approval

This study was approved by the University of Southern California IRB and was performed according to institutional guidelines.

### Informed consent

Per institutional guidelines, informed consent from participants was not required for this retrospective analysis of deidentified data.

## Results

**Figure 3:**
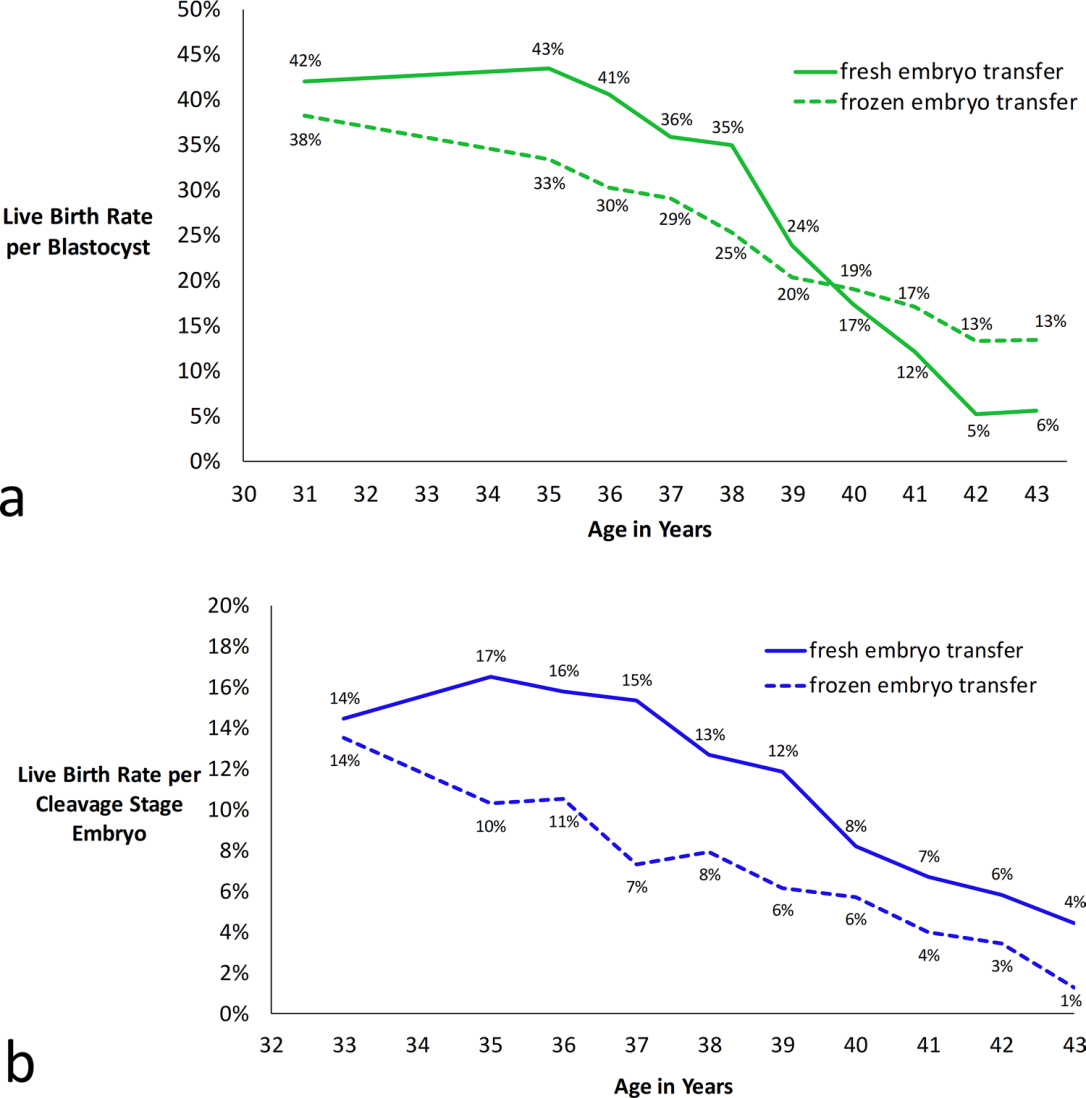
Live Birth Rate per Embryo for (**a**) Blastocyst and (**b**) Cleavage Stage Embryos. 5-year moving groups are used to calculate live birth rates for ages 35 to 43.

**Figure 4:**
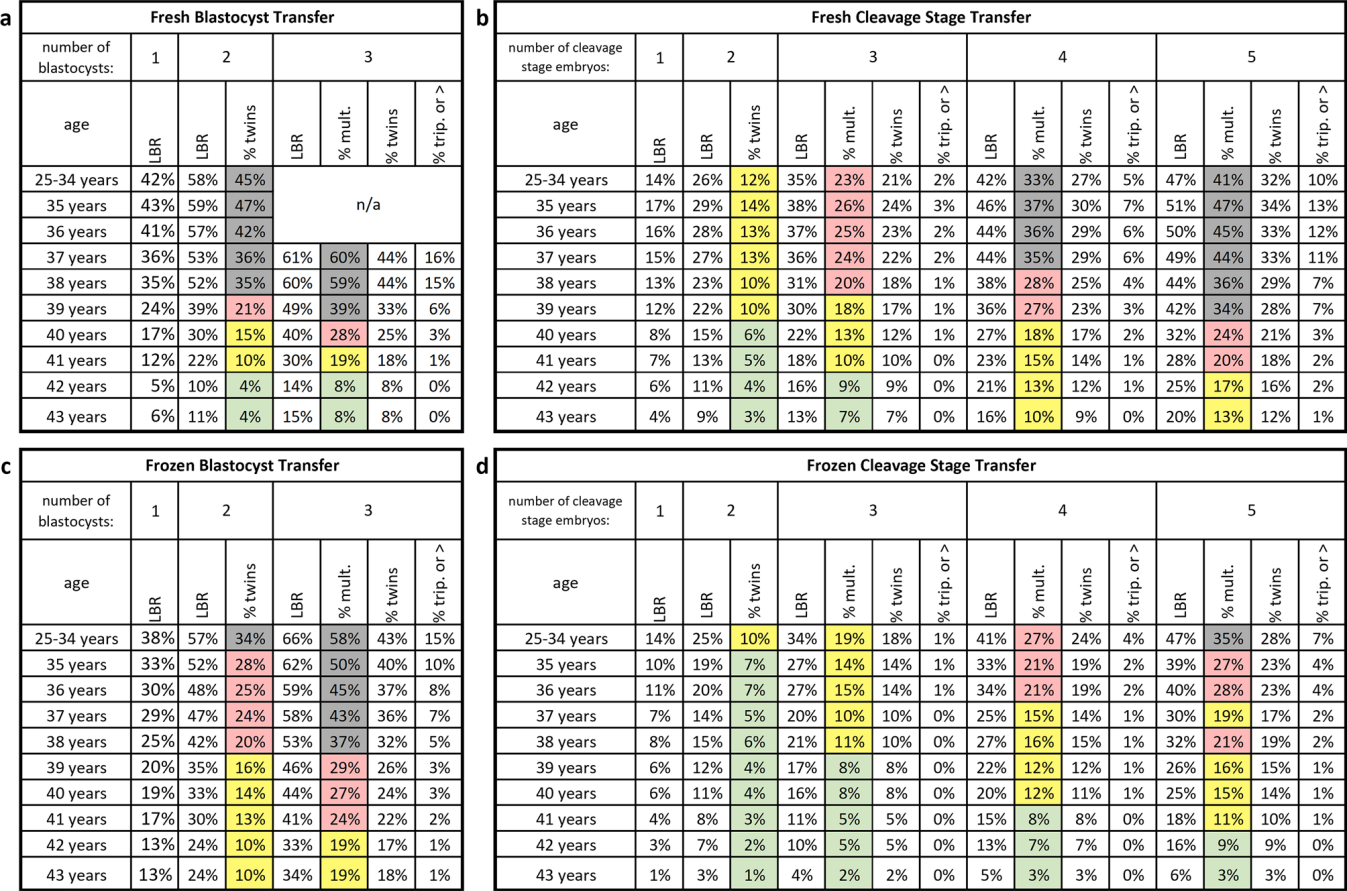
Predicted outcomes for (**a**) Fresh Blastocyst, (**b**) Fresh Cleavage Stage, (**c**) Frozen Blastocyst, and (**d**) Frozen Cleavage Stage Embryo Transfers. The multiples column results are shaded green, yellow, red, or gray to indicate the risk of multiples at delivery (0–9%, 10–19%, 20–29%, or ≥ 30% respectively). LBR, total live birth rate per embryo transfer; % mult., percentage of live deliveries that are multiples; % twins, percentage of live deliveries that are twin deliveries; % trip. or > , percentage of live deliveries that are triplets or greater.

The live birth rate per embryo varied from as high as 43% for fresh blastocysts in the 35-year-old age group to as low as 1% for frozen cleavage stage embryos in the 43-year-old age group as shown in Fig. 3a, b. 95% CIs and best fit ongoing pregnancy rates are shown in Supplemental Table S2. The best fit UNI was 0.68 for fresh embryo transfers and 0.75 for frozen embryo transfers (Supplemental Fig. S3). Best fit values of UNI for different groupings of embryo transfers and based on ongoing pregnancy rates are shown in Supplemental Table S3. Based on these results and the logic in Fig. 2b, predicted embryo transfer outcomes are shown in Fig. 4 with the multiples column shaded green, yellow, red, or gray to indicate the risk of multiples at delivery (0–9%, 10–19%, 20–29%, or ≥ 30% respectively).

## Discussion

The methods used in this analysis differ from those of previous models through the use of moving age groups, estimation of LBRs using linear algebra, a universal factors fraction, automated data processing and model validation through custom computer programs in MATLAB, and a simplified presentation of the end results in tables that can be used quickly in clinic (Fig. 4).

In this analysis we introduce a “moving groups” analysis when determining embryo live birth rates. We group patients less than 35 years old into one age group because clinically we expect similar live birth rates and there were small numbers of patients in this group. For ages 35 through 43 years, 5-year moving groups centered on the age of interest best smoothed out random noise without over-smoothing. We assume that lower live birth rates associated with older patients in a group will balance out higher live birth rates from younger patients.

We base our estimation of quality (live birth rate per embryo) on characteristics that can be determined prior to oocyte retrieval and can be easily compared between clinics: oocyte age at retrieval, embryo stage (cleavage or blastocyst) and cycle type (fresh or frozen). Including embryo morphology in a predictive model increases the complexity of validating the universal factors model and is more suited for a separate focused study. We have recently analyzed the association of cleavage stage and blastocyst morphology with live birth and described how morphology can be incorporated into the predictive model^19,21^. Using a live birth rate assuming average embryo morphologic quality seems reasonable and is clinically useful for patient counseling both before retrieval and prior to transfer. We use a system of equations to determine best fit live birth rates by preference mostly because this approach is needed to evaluate data when embryos of different morphologic qualities are transferred concurrently.

We expand on the concept of “uterine receptivity” and present a new method for its estimation. In 1983 Speirs denoted uterine factors by a capital “U” and described the concept as the “upper limit of the overall pregnancy rate if a large number of healthy embryos were transferred”^4^. We use the same upper limit definition provided by Speirs but prefer the terminology “universal factors fraction” denoted by “UNI” to unambiguously refer to all factors that impact all embryos transferred concurrently. Universal factors include endometrial receptivity, endometritis, anatomical uterine abnormalities, embryo transfer technique, uterine location of transferred embryos, embryo culture conditions, air quality, maternal medical comorbidities, parental genetic abnormalities, autoimmunity, and other factors.

The universal factors fraction can only be calculated through indirect measures such as outcomes of multiple embryo transfers. During transfer of multiple embryos with known live birth rates per embryo, a UNI closer to 1 will result in higher rates of singleton births and lower rates of twin births (Fig. 2b and Supplemental Fig. S3). In our analysis we decided to group fresh (UNI = 0.68) and frozen (UNI = 0.75) embryo transfers together when calculating the UNI and performing the validation studies. This grouping is based on current thought of uterine factors being more favorable in frozen transfers. For the frozen cleavage stage embryos there were only 88 total embryo transfers and our actual outcomes for this subgroup did not fit with a UNI value of 1 or less so the UNI value could not be determined for this subgroup (Supplemental Table S3). While this could be interpreted to mean that the implantation of one embryo impeded the implantation of additional embryos, a more likely explanation is that this is a result of random variation. We use best fit values of UNI since there was not enough data to determine UNI with any significant confidence. Approximately 5,000 transfers of multiple embryos are required to calculate UNI with a 95% CI of ± 0.05 ^20^. Our calculated universal factors fraction of 0.68, or 68%, for fresh embryo transfers is similar to the value estimated by Speirs in 1983 of “about 70%”^4^.

If we do not take universal factors into account and assume each embryo implants independently as if universal factors are always favorable (UNI = 1, Fig. 2a) then our observed rate of twins is higher than predicted and the observed rate of singleton delivery is lower than predicted for fresh transfers (Supplemental Fig. S1D). This is corrected when we apply a best fit value of UNI to the same data (Supplemental Fig. S1B). This is less pronounced for frozen embryo transfers where the UNI value in our model is greater suggesting that universal factors are more favorable overall (Supplemental Fig. S1F and S1H).

Instead of presenting predicted outcomes by complicated equations involving exponentials and factorials, computer programs are used make the calculations and display the results in an easy-to-read format (Fig. 4)^20^. This incorporates the logic of concepts such as the binomial formula (which mathematically describes all possible combinations of a given number of embryos) and the universal factors fraction (Fig. 2b). The predicted outcomes in Fig. 4 are similar to those observed by other investigators^22–24^. A simplified form of this model could be adopted by other clinics. Live birth rates for 5-year moving groups (centered on the age of interest) could be calculated simply as live births divided by number of embryos transferred. A universal factors fraction of 0.70 could be used for both fresh and frozen transfers. Predicted outcomes for different combinations of average live birth rate per embryo and number of embryos transferred are given for UNI = 0.70 in Supplemental Fig. S4 along with an example table which can be used for other clinics to easily analyze their own data.

## Limitations

Due to the number of other factors considered and a limited dataset, we were unable to incorporate embryo morphology into this prediction model. The environment for embryo implantation may vary from patient to patient based on variables other than embryo stage and transfer cycle type (fresh or frozen) which are discussed here. While our model predicts rates of higher order multiples, our dataset contained only one case of triplet delivery and therefore we were not able evaluate use of this model for predicting higher order multiples. This model is meant to account only for dizygotic twinning. In clinical use, patients should be counseled that monozygotic twinning could add an additional 1–2% to the predicted rate of multiple gestation after transfer of a single embryo or multiple embryos. It is uncertain how applicable results from a single IVF clinic are to other clinics.

## Conclusion

We describe and validate a model for predicting live singleton and twin delivery rates after cleavage and blastocyst stage embryo transfers. We use a system of equations approach to concurrently analyze data from both single and multiple embryo transfers. Our quantitative estimation of universal factors is close to that estimated by Speirs in 1983. New datasets can be analyzed with these methods or a simplified version in hours. The model predictions are reported in easy-to-read tables that can be used to guide the number of embryos to transfer to maximize pregnancy rates while limiting multiple gestations.

## Supplementary Information

Supplementary Information.

## Data Availability

The MATLAB code used to generate all of the data for figures and tables in this manuscript is being made available through Mendeley Data at http://dx.doi.org/10.17632/mg8b5nv3g5.
