# Supplementary material for: A validated model for predicting live birth after embryo transfer
Source: Sci Rep. 2021 May 24;11:10800. doi: 10.1038/s41598-021-90254-y (PMC8144418; doi:10.1038/s41598-021-90254-y)
Supplement: Supplementary file 1 — Supplementary Information. [file 41598_2021_90254_MOESM1_ESM.pdf]

Supplemental Material

A Validated Model for Predicting Live Birth After Embyo Transfer

Michael S. Awadalla, M.D., Kristin A. Bendikson, M.D., Jacqueline R. Ho, M.D., Lynda K. McGinnis, Ph.D., Ali Ahmady, Ph.D.

Table of Contents

Supplemental Methods 2
MATLAB Code 2
Calculation of 95% Confidence Intervals 2
In Vitro Fertilization Protocols 2
Ovarian Stimulation 2
Oocyte Collection, IVF, and ICSI 2
Embryo Culture 3
Fresh Embryo Transfer 3
Vitrification Technique 3
Frozen Embryo Transfer 3
Supplemental Figures 4
Supplemental Figure S1: Predicted and Observed Rates of Singleton, Twin, and Total Live Birth in 10-Fold Cross-Validation Study 4
Supplemental Figure S2: Quantile-Quantile Plots of Standardized Differences Between Observed and Expected Outcomes in 10-Fold Cross-Validation Study. 6
Supplemental Figure S3: Best Fit Universal Factors Fraction (UNI) for Fresh and Frozen Embryo Transfers. 7
Supplemental Figure S4: Simplified Model for Fitting Individual Clinic Data. 8
Supplemental Tables 9
Supplemental Table S1: Demographics and Cycle Characteristics. 9
Supplemental Table S2: Best Fit Live Birth and Ongoing Pregnancy Rates Per Embryo by Age Group With 95% CIs. 10
Supplemental Table S3: Best Fit Universal Factors Fraction (UNI) Based on Live Birth and Ongoing Pregnancy. 11
References 11

Supplemental Methods

MATLAB Code

For reproducibility, the MATLAB code used to generate all of the data for figures and tables in this manuscript is being made available through Mendeley Data[1]. All of the data analysis and pertinent figure generation can be performed by running the file "generate_data_and_figures.m" with MATLAB. Descriptions for each of the 12 included MATLAB programs are included in the comments section at the beginning of each program.

Calculation of 95% Confidence Intervals

Bootstrapping was used to simulate embryo transfers based on the logic in Figure 2B to determine 95% confidence intervals (CIs) for the live birth rates. A custom computer program in MATLAB was used to perform the bootstrapping. A random number generator was used to simulate universal factors that affect all embryos such that if universal factors were not favorable no embryos implanted. If factors were favorable, embryos were assumed to implant independently with a live birth rate of $\frac{\mathrm{LBR}}{\mathrm{UNI}}$ where UNI denotes a universal factors fraction and LBR denotes the overall live birth rate of an embryo. A universal factors fraction of 0.70 was used to model favorable universal factors 70% of the time for combined analysis of fresh and frozen embryo transfers. For analysis of fresh embryo transfers a value of 0.68 was used and for frozen embryo transfers a value of 0.75 was used. These are the approximate values from our embryo transfer data (Table S3) and that of others[2]. If the uterine factors were favorable, random number generation was again used to determine if each embryo would implant or not based on the logic in Figure 2B which is an example of logic for a double embryo transfer. For each group of embryo transfers the outcomes were simulated 100,000 times and for each simulation best fit live birth rates were calculated to create a probability distribution of predicted live birth rates. 95% CIs for the live birth rates were taken from the probability distribution determined by the simulation. The MATLAB code for the computer simulation is being provided through Mendeley Data and the analysis can be performed by running the "generate_data_and_figures.m" file[1]. The 95% CIs for live birth rates are given in supplemental table 2 for each age group.

In Vitro Fertilization Protocols

Ovarian Stimulation

Ovarian stimulation was performed with recombinant human follicle stimulating hormone (FSH) 75-300IU SC daily (Follistim: Merck, Kenilworth, NJ; or Gonal-f: EMD Serono, Rockland, MD) and/or human menopausal gonadotropins (hMG) 75-150IU SC daily (Menopur: Ferring Pharmaceuticals, Parsippany, NJ). Pituitary suppression was achieved through the use of gonadotropin releasing hormone (GnRH) antagonist, GnRH agonist suppression, or GnRH flare suppression protocols. In antagonist cycles the GnRH antagonist (0.25mg SC ganirelix acetate daily or 0.25mg SC cetrorelix acetate daily) was started when the lead follicle reached 14mm in diameter at which time LH receptor stimulation was begun with SC hMG and/or 100IU SC human chorionic gonadotropin (hCG) daily. When two lead follicles reached 18mm in diameter or three follicles reached 17mm in diameter patients received a trigger medication for oocyte maturation and the GnRH antagonist was discontinued. 5,000IU hCG (or 10,000IU HCG for patients with a weight of 200lbs or greater) was used for the trigger in GnRH agonist and GnRH flare protocols. With GnRH antagonist protocols hCG, leuprolide (4mg SC every 12 hours for 2 doses), or a hCG (2,500IU SC) and leuprolide co-trigger was used. Follicular aspiration was performed 34-36 hours after the trigger administration. Doxycycline 100mg PO twice a day was prescribed starting in the morning prior to the follicular aspiration and continued for 3 days for patients freezing embryos and until the morning of embryo transfer for patients receiving a fresh embryo transfer.

Oocyte Collection, IVF, and ICSI

Oocyte cumulus complexes were identified in the follicular fluid with an Olympus SZX10 microscope and transferred to oocyte collection dishes in Multipurpose Handling Medium with Gentamicin (MHM, Irvine Scientific, Santa Ana, CA). Oocytes were placed in MHM before insemination or ICSI. IVF insemination was carried out 4-6 hours after the retrieval with each droplet being inseminated with 50,000 motile sperm. ICSI was performed on mature MII oocytes after removal of cumulus coronal cells. Removal of cumulus coronal cells was performed by exposure to hyaluronidase for 60 seconds followed by transfer to MHM and pipetting with 300µm, 170µm, 140µm, and 130µm diameter pipettes. ICSI was performed with an Olympus IX73 microscope microinjector (Olympus Corporation, Shinjuku City, Japan) with a heated stage (Tokai Hit, Shizuoka, Japan) and Narishige micromanipulator (Narishige, Tokyo, Japan). At 16-18 hours post insemination or ICSI a fertilization check was performed.

Embryo Culture
Embryos were cultured in 15 µL drops of Continuous Single Culture Complete with Gentamicin and Human Serum Albumin (HSA) media (CSCM-C, Irvine Scientific, Santa Ana, CA) which was replaced with fresh media on day 4 of culture. Embryos were cultured in 38Special GPS Dishes (Life Global, Guilford, Connecticut, USA) overlaid with Liteoil (Life Global) for 3 days for cleavage stage embryos and 4-7 days for blastocysts. The incubator conditions were set to temperature 37.0ºC, 5% O_2, 8% CO_2, and 87% N_2. The % CO_2 was titrated to maintain a culture media pH of 7.2-7.5 with a preferred pH range of 7.25-7.35. The embryos were cultured in either a Miri tabletop incubator (ESCO Medical, Changi, Singapore) or HERAcell 150i incubator (Thermo Scientific, Waltham, MA).

Fresh Embryo Transfer
After follicular aspiration patients were prescribed 2mg estradiol PO twice daily until a positive serum HCG and 200mg micronized vaginal progesterone twice daily until 10 weeks gestation.

Vitrification Technique
Blastocysts of grade 1CC or higher and cleavage stage embryos with 6-10 cells on day 3 were considered for vitrification. Blastocyst vitrification was performed according to the I.C.E. Blastocyst Vitrification Protocol (Innovative Cryo Enterprises LLC, Linden, New Jersey). Cryotop vitrification device (KitaZato, Tokyo, Japan) was used for blastocyst vitrification. Cryotip device (Irvine Scientific, Santa Ana, CA) was used for cleavage stage embryo vitrification. Prior to vitrification, blastocysts were collapsed with laser pulses directed at a thin portion of the trophectoderm away from the inner cell mass to prevent ice crystal formation. Vitrification was performed 10-30 minutes after the embryo was collapsed.

Frozen Embryo Transfer
The majority of frozen embryo transfers occurred in programmed cycles. Starting on day 2 of the menses a baseline transvaginal ultrasound was performed and 2mg oral estradiol was administered twice a day for 6 days then three times a day until embryo transfer. On approximately day 11 of estradiol a serum progesterone and endometrial thickness by transvaginal ultrasound were measured. Cycles were cancelled if the progesterone was greater than or equal to 1.5 ng/mL. Additional days of estradiol were prescribed if the endometrial thickness was less than 7.0mm. If the serum progesterone was less than 1.5 ng/mL and the endometrial thickness was equal to or greater than 7.0mm, then IM and vaginal progesterone was started. IM progesterone was started with 50mg progesterone in ethyl oleate starting at 9PM (day 1 of progesterone). Subsequent days of progesterone consisted of IM progesterone at 9AM and 200mg micronized progesterone vaginally at 1PM and 9PM. Cleavage stage transfers were scheduled on day 4 of progesterone (approximately 60 hours after the first IM progesterone dose) and blastocyst transfers were scheduled on day 6 of progesterone (approximately 108 hours after the first IM progesterone dose). This timing was adjusted to the implantation window if a patient had previously had an endometrial biopsy for receptivity testing. Doxycycline 100mg orally was prescribed twice a day starting in the morning the day prior to the embryo transfer for a total of three doses. After embryo transfer, patients were prescribed oral estradiol 2mg twice a day, 50mg IM progesterone in ethyl oleate once daily, and 200mg micronized progesterone vaginally twice a day for 13 weeks. The first serum HCG was measured 11 days after cleavage stage transfers and 9 days after blastocyst transfers.

Supplemental Figures

Supplemental Figure S1: Predicted and Observed Rates of Singleton, Twin, and Total Live Birth in 10-Fold Cross-Validation Study.

Validation study results for fresh embryo transfers are shown in Panels A and B for the logic in Figure 2B and in Panels C and D for the logic in Figure 2A for comparison. Result for frozen embryo transfers are shown in Panels E and F for the logic in Figure 2B and in Panels G and H for the logic in Figure 2A.

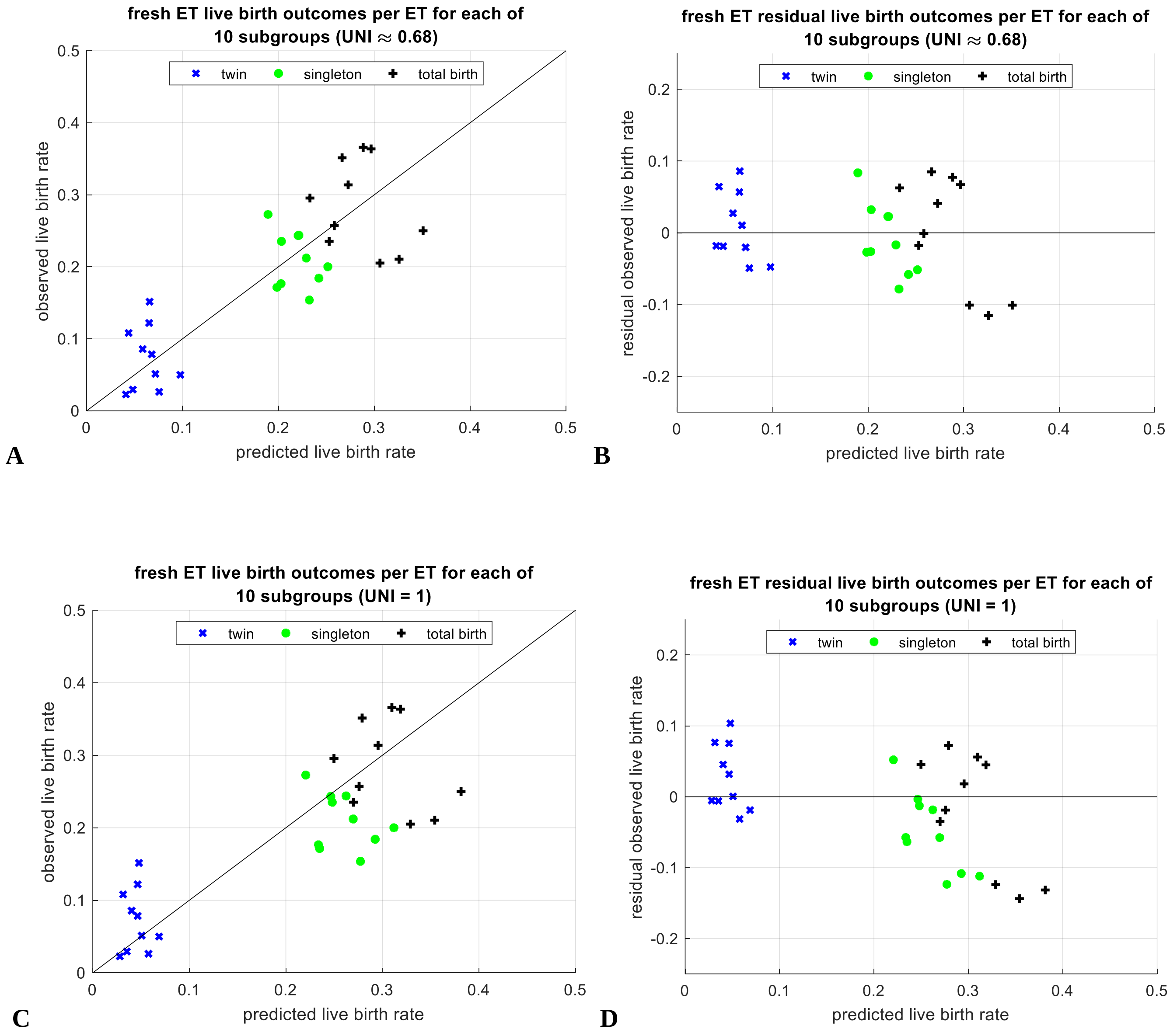

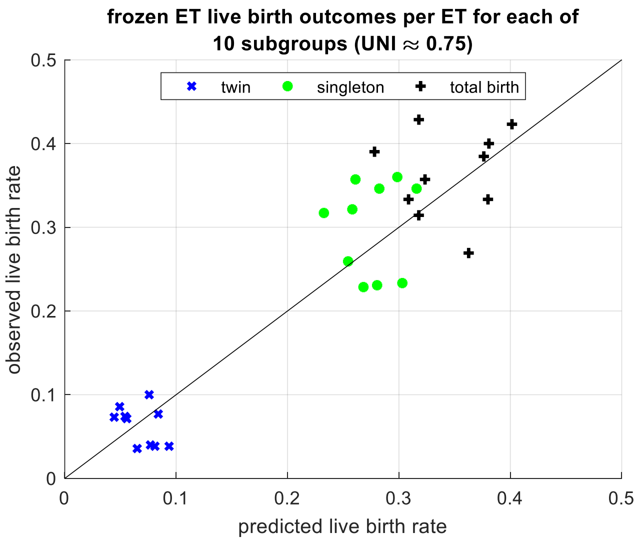
frozen ET live birth outcomes per ET for each of
10 subgroups (UNI ≈ 0.75)
twin
singleton
total birth
observed live birth rate
predicted live birth rate
0
0.1
0.2
0.3
0.4
0.5

E

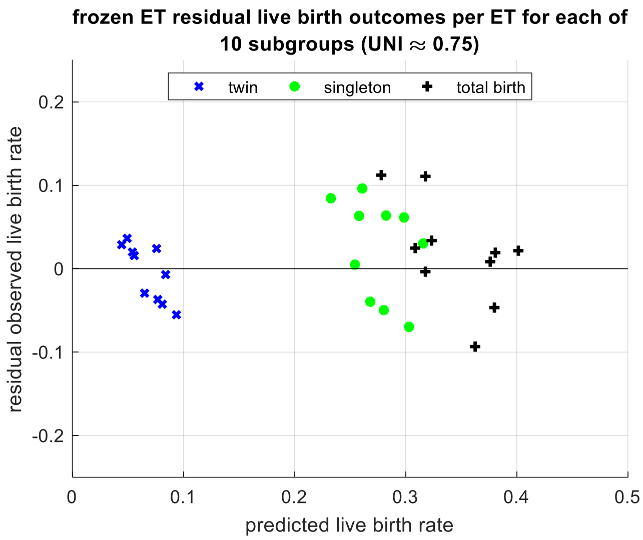
frozen ET residual live birth outcomes per ET for each of
10 subgroups (UNI ≈ 0.75)
twin
singleton
total birth
residual observed live birth rate
predicted live birth rate
0.2
0.1
0
-0.1
-0.2
0
0.1
0.2
0.3
0.4
0.5

F

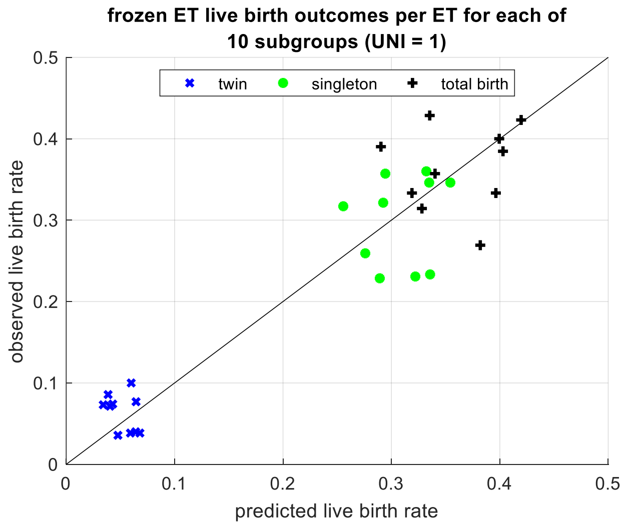
frozen ET live birth outcomes per ET for each of
10 subgroups (UNI = 1)
twin
singleton
total birth
observed live birth rate
predicted live birth rate
0
0.1
0.2
0.3
0.4
0.5

G

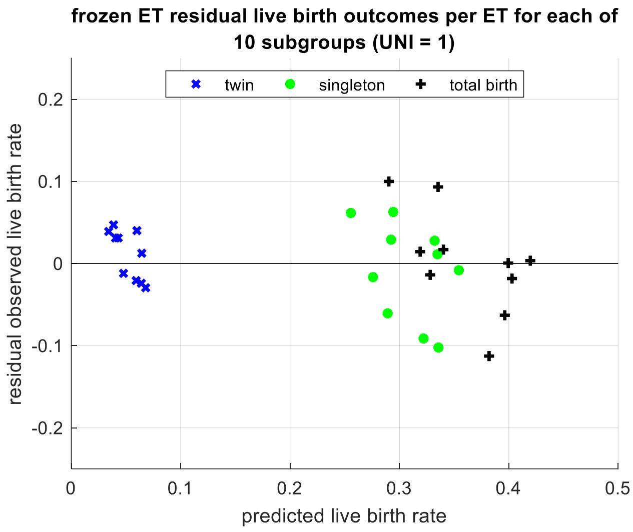
frozen ET residual live birth outcomes per ET for each of
10 subgroups (UNI = 1)
twin
singleton
total birth
residual observed live birth rate
predicted live birth rate
0.2
0.1
0
-0.1
-0.2
0
0.1
0.2
0.3
0.4
0.5

H

Supplemental Figure S2: Quantile-Quantile Plots of Standardized Differences Between Observed and Expected Outcomes in 10-Fold Cross-Validation Study.

The mean standardized errors were not significantly different from zero for the fresh transfer predicted singleton delivery rate per transfer (p = 0.73, Panel B) or twin delivery rate per transfer (p = 0.40, Panel C). The mean standardized errors for frozen transfers were not different from zero for singleton delivery rate per transfer (p = 0.32, Panel E) or twin delivery rate (p = 1.00, Panel F). Panels A and D are the plots for total live birth.

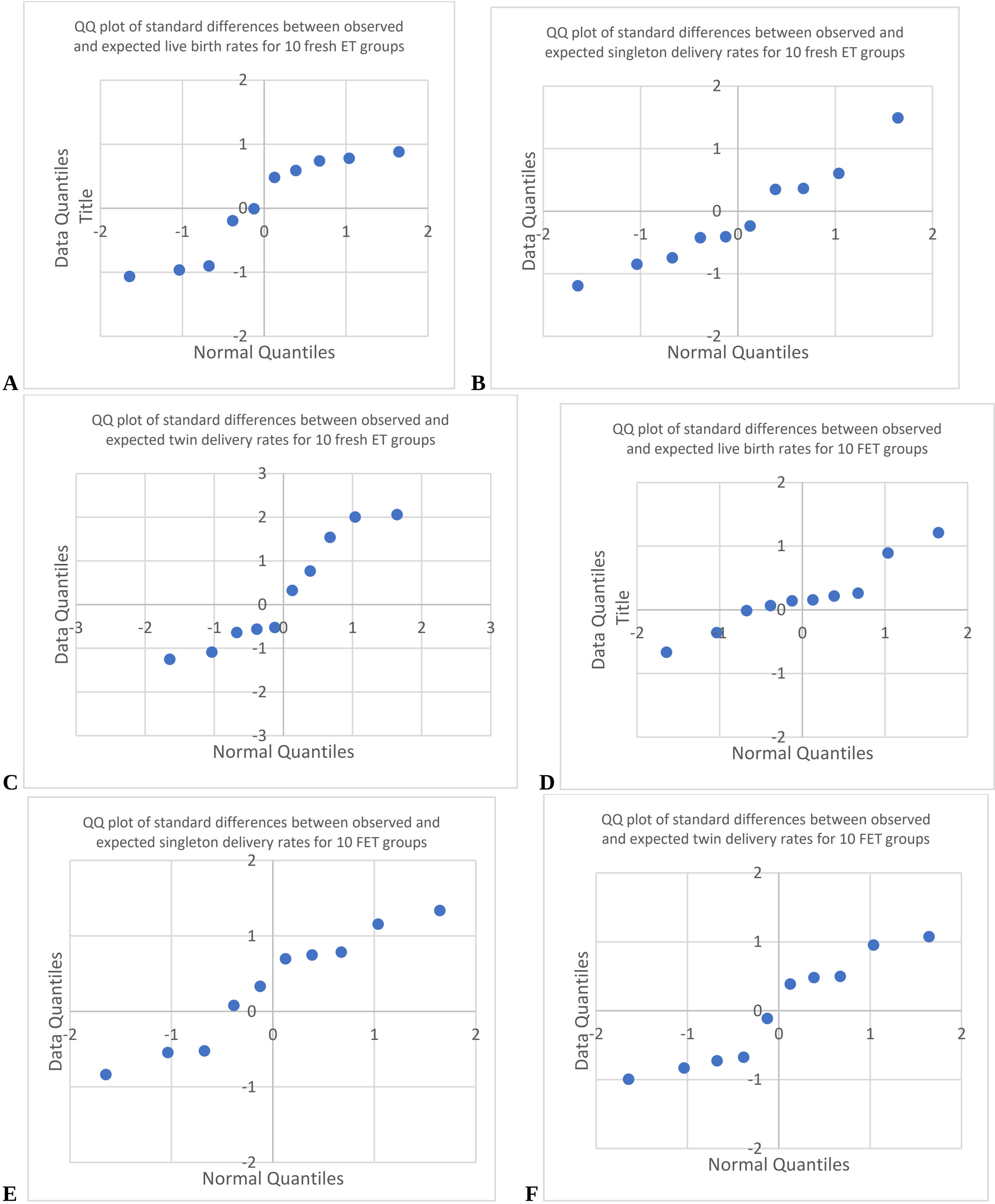

Supplemental Figure S3: Best Fit Universal Factors Fraction (UNI) for Fresh and Frozen Embryo Transfers. The best fit UNI for fresh embryo transfers was 0.68 (Panel A) and the best fit UNI for frozen embryo transfers was 0.75 (Panel B).

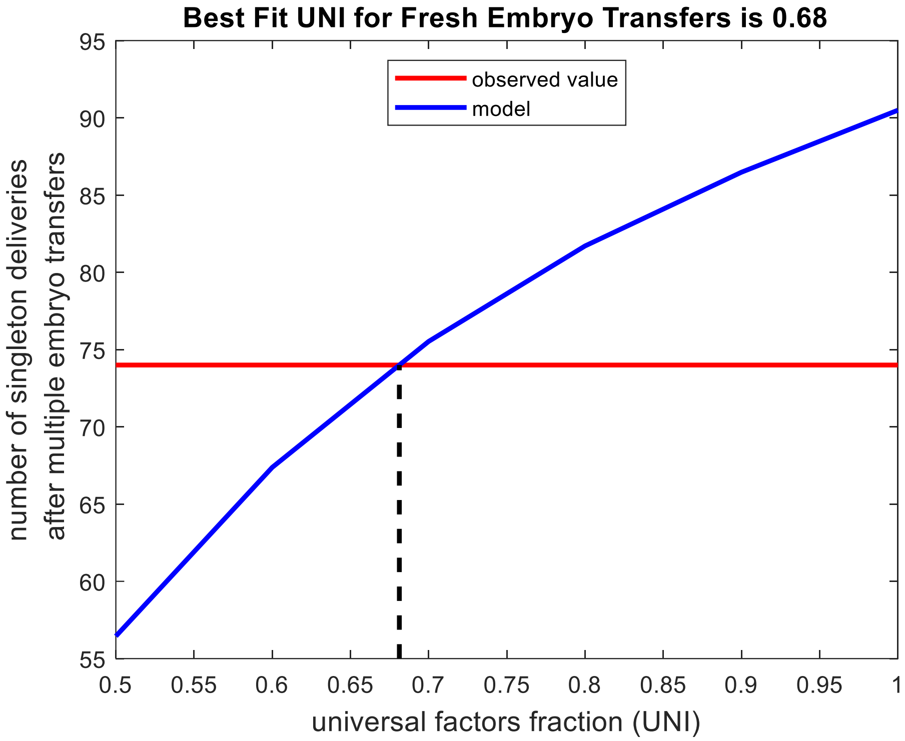

A

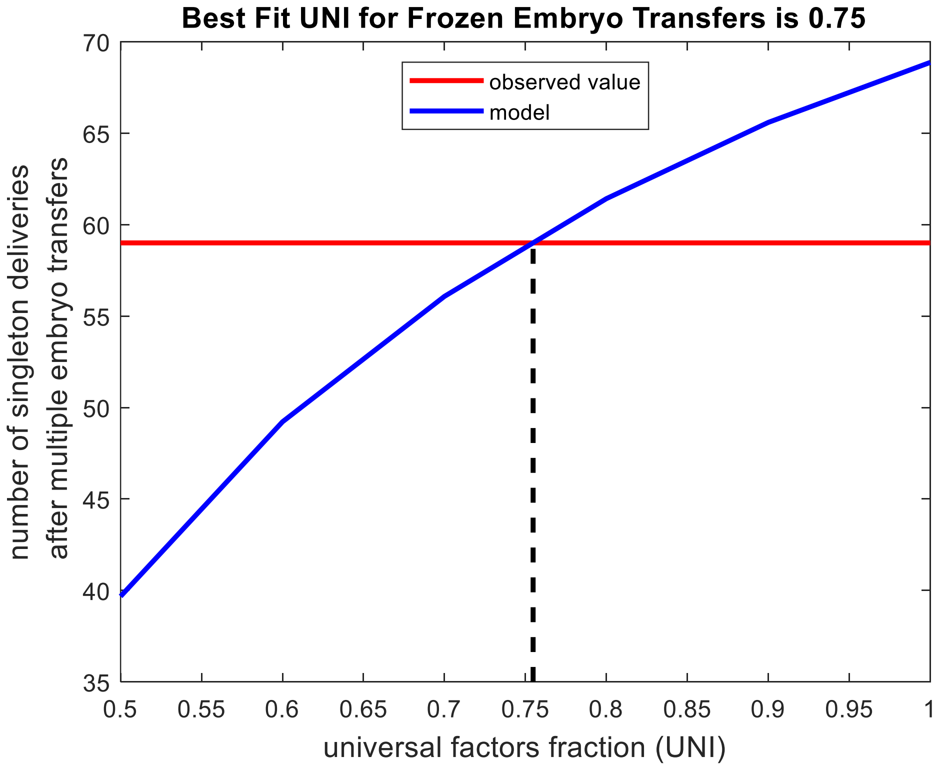

B

Supplemental Figure S4: Simplified Model for Fitting Individual Clinic Data.

A. Predicted Transfer Outcomes by Average Live Birth Rate per Embryo and Number of Embryos for Universal Factors Fraction of 0.70.

number of embryos:	1	2		3				4				5				6				7			
outcomes:	LBR	LBR	% twins	LBR	% mult.	% twins	% trip. or >	LBR	% mult.	% twins	% trip. or >	LBR	% mult.	% twins	% trip. or >	LBR	% mult.	% twins	% trip. or >	LBR	% mult.	% twins	% trip. or >
	50%																						
	48%																						
	46%	62%	49%																				
	44%	60%	46%																				
	42%	59%	43%																				
	40%	57%	40%																				
	38%	55%	37%																				
	36%	53%	35%																				
	34%	51%	32%																				
	32%	49%	30%																				
	30%	47%	27%	57%	48%	39%	10%																
	28%	45%	25%	55%	45%	37%	8%																
average live birth rate per embryo	26%	42%	23%	53%	41%	35%	7%																
	24%	40%	21%	50%	38%	32%	6%																
	22%	37%	19%	47%	35%	30%	5%	55%	48%	36%	12%												
	20%	34%	17%	44%	31%	28%	4%	52%	44%	34%	10%												
	18%	31%	15%	41%	28%	25%	3%	49%	39%	31%	8%	54%	49%	35%	14%								
	16%	28%	13%	38%	25%	22%	2%	45%	35%	29%	6%	51%	44%	33%	11%								
	14%	25%	11%	34%	21%	20%	2%	41%	31%	26%	5%	47%	39%	30%	9%	52%	47%	33%	13%				
	12%	22%	9%	30%	18%	17%	1%	37%	26%	23%	3%	43%	34%	27%	6%	47%	41%	31%	10%	51%	47%	33%	14%
	10%	19%	8%	26%	15%	14%	1%	32%	22%	20%	2%	38%	28%	24%	4%	42%	34%	27%	7%	46%	40%	30%	10%
	8%	15%	6%	21%	12%	11%	0%	27%	17%	16%	1%	32%	23%	20%	3%	36%	28%	23%	4%	40%	33%	26%	6%
	6%	11%	4%	17%	9%	9%	0%	21%	13%	12%	1%	25%	17%	16%	2%	29%	21%	19%	2%	33%	25%	21%	4%
	4%	8%	3%	11%	6%	6%	0%	15%	9%	8%	0%	18%	11%	11%	1%	21%	14%	13%	1%	24%	17%	15%	2%
	2%	4%	1%	6%	3%	3%	0%	8%	4%	4%	0%	9%	6%	6%	0%	11%	7%	7%	0%	13%	8%	8%	0%

Greater than 50% of live deliveries are predicted to be multiples.

The multiples column results are shaded green, yellow, red, or gray to indicate the risk of multiples at delivery (0-9%, 10-19%, 20-29%, or ≥ 30% respectively). LBR = total live birth rate per embryo transfer; % mult. = percentage of live deliveries that are multiples; % twins = percentage of live deliveries that are twin deliveries; % trip. or > = percentage of live deliveries that are triplets or greater.

B. Sample Table for Simple Calculation of Live Birth Rate per Embryo for Different Age Groups to be Used with Panel A.

	Cleavage Stage Transfers					Blastocyst Stage Transfers				
age	embryos transferred	live births	total embryos transferred in group	total live births in group	live birth rate	embryos transferred	live births	total embryos transferred in group	total live births in group	live birth rate
30										
31										
32										
33										
34										
35										
36										
37										
38										
39										
40										
41										
42										
43										

Supplemental Tables

Supplemental Table S1: Demographics and Cycle Characteristics.

	Mean (SD)
**Age**	37.5 (4.3)
**BMI**	24.7 (5.1)
**Race/Ethnicity**	
White	40.2%
Asian	30.4%
Hispanic	15.9%
African American	5.3%
American Indian	0.2%
Multiple	5.0%
Unknown	3.1%
**Transfer Type**	
Fresh cleavage	43.7%
Frozen cleavage	12.9%
Fresh blastocyst	13.6%
Frozen blastocyst	29.8%
**Stimulation Cycle**	
GnRH Antagonist	56.3 %
GnRH Flare	36.8 %
GnRH Agonist Suppression	6.9 %

Supplemental Table S2: Best Fit Live Birth and Ongoing Pregnancy Rates Per Embryo by Age Group With 95% CIs.

	< 35 years	35 years	36 years	37 years	38 years	39 years	40 years	41 years	42 years	43 years
**Blastocyst Live Birth Rate**										
All blastocysts	39% (32-47%)	37% (30-44%)	34% (27-41%)	31% (25-38%)	28% (21-35%)	21% (15-28%)	19% (13-25%)	16% (9-23%)	11% (5-19%)	11% (4-20%)
Fresh blastocysts	42% (28-56%)	43% (30-57%)	41% (28-53%)	36% (24-48%)	35% (22-49%)	24% (11-39%)	17% (5-32%)	12% (1-27%)	5% (0-16%)	6% (0-17%)
FET blastocysts	38% (29-48%)	33% (25-42%)	30% (23-38%)	29% (21-37%)	25% (18-33%)	20% (14-28%)	19% (12-27%)	17% (9-26%)	13% (5-22%)	13% (4-25%)
**Cleavage Stage Embryo Live Birth Rate**										
All cleavage stage	14% (7-22%)	15% (11-20%)	15% (11-19%)	14% (10-18%)	12% (9-15%)	11% (8-14%)	8% (5-10%)	6% (4-9%)	5% (4-8%)	4% (2-6%)
Fresh cleavage stage	14% (6-24%)	17% (11-22%)	16% (11-21%)	15% (11-20%)	13% (9-17%)	12% (8-16%)	8% (5-11%)	7% (4-9%)	6% (4-8%)	4% (2-7%)
FET cleavage stage	14% (3-27%)	10% (3-19%)	11% (4-19%)	7% (2-14%)	8% (3-14%)	6% (2-12%)	6% (2-11%)	4% (1-9%)	3% (0-7%)	1% (0-4%)
**Blastocyst Ongoing Pregnancy Rate**										
All blastocysts	45% (36-53%)	42% (34-50%)	40% (32-47%)	38% (31-46%)	35% (28-43%)	28% (21-36%)	27% (19-34%)	24% (16-32%)	18% (10-28%)	17% (8-27%)
Fresh blastocysts	50% (35-65%)	48% (34-61%)	46% (33-59%)	41% (28-54%)	40% (25-55%)	33% (17-50%)	26% (11-42%)	18% (4-34%)	9% (0-22%)	9% (0-26%)
FET blastocysts	42% (32-51%)	39% (30-48%)	37% (28-45%)	37% (29-46%)	34% (25-42%)	27% (19-35%)	27% (18-35%)	26% (16-36%)	21% (11-32%)	20% (9-33%)
**Cleavage Stage Embryo Ongoing Pregnancy Rate**										
All cleavage stage	15% (8-24%)	18% (13-23%)	17% (13-22%)	16% (12-21%)	14% (11-18%)	13% (10-16%)	9% (7-12%)	8% (5-10%)	7% (4-9%)	5% (3-7%)
Fresh cleavage stage	14% (6-24%)	19% (13-25%)	18% (13-24%)	18% (13-23%)	15% (11-19%)	13% (10-17%)	9% (6-13%)	7% (5-10%)	7% (4-9%)	5% (3-7%)
FET cleavage stage	18% (4-32%)	13% (5-23%)	13% (5-22%)	11% (4-19%)	11% (5-18%)	10% (4-18%)	10% (4-16%)	9% (3-15%)	7% (2-12%)	5% (1-10%)

5-year moving groups are used for ages 35 to 43.

Supplemental Table S3: Best Fit Universal Factors Fraction (UNI) Based on Live Birth and Ongoing Pregnancy.

	Fresh embryo transfer	Frozen embryo transfer	Fresh and frozen combined
**Best Fit UNI Based on Live Birth**			
Cleavage stage	0.61	n/a*	0.69
Blastocyst stage	0.74	0.68	0.69
Cleavage and blastocyst combined	**0.68****	**0.75****	0.69
**Best Fit UNI Based on Ongoing Pregnancy**			
Cleavage stage	0.70	n/a*	0.84
Blastocyst stage	0.78	0.79	0.78
Cleavage and blastocyst combined	0.75	0.88	0.79

The universal factors fraction is calculated as a best fit value based on number of singleton live births that resulted from multiple embryo transfers.

* This value could not be determined as results were not consistent with a best fit value of 1 or less.

** The best fit UNI values for live birth after fresh and frozen embryo transfers (for cleavage and blastocyst stage combined) are used in the main analysis and validation study.

References

1. Awadalla, M. S. MATLAB Code for ‘A Validated Model for Predicting Live Birth After Embryo Transfer.’ Mendeley Data, V1 2020. http://dx.doi.org/10.17632/mg8b5nv3g5.1.

2. Speirs, A. L., Lopata, A., Gronow, M. J., Kellow, G. N. & Johnston, W. I. Analysis of the benefits and risks of multiple embryo transfer. *Fertil. Steril.* **39**, 468–471 (1983).
